# SARS‐CoV‐2 and omicron variant detection with a high selectivity, sensitivity, and low‐cost silicon bio‐nanosensor

**DOI:** 10.1002/nano.202200188

**Published:** 2022-12-29

**Authors:** Antonio Alessio Leonardi, Emanuele Luigi Sciuto, Maria José Lo Faro, Barbara Fazio, Maria Giovanna Rizzo, Giovanna Calabrese, Luca Francioso, Rosaria Picca, Francesco Nastasi, Giuseppe Mancuso, Corrado Spinella, Wolfgang Knoll, Alessia Irrera, Sabrina Conoci

**Affiliations:** ^1^ Dipartimento di Fisica e Astronomia “Ettore Majorana” Università degli studi di Catania Catania Italy; ^2^ CNR‐IMM Catania Università Istituto per la Microelettronica e Microsistemi Catania Italy; ^3^ Lab SENS Beyond Nano CNR Messina Italy; ^4^ Dipartimento di Scienze Chimiche Biologiche, Farmaceutiche, ed Ambientali Università degli studi di Messina Messina Italy; ^5^ CNR‐IMM Istituto per la Microelettronica e Microsistemi Via Monteroni University Campus Lecce Italy; ^6^ Dipartimento di Chimica Università degli studi di Bari Bari Italy; ^7^ Dipartimento di Patologia Umana dell'adulto e dell'età evolutiva Gaetano Barresi Università degli studi Messina Gazzi (Me) Italy; ^8^ CNR‐IMM Istituto per la Microelettronica e Microsistemi Zona Industriale Catania Italy; ^9^ Department of Scientific Coordination and Management Danube Private University Krems Austria; ^10^ Dipartimento di Chimica "G. Ciamician" Università degli studi di Bologna Bologna Italy

**Keywords:** biosensor, omicron, optical sensing, SARS‐CoV‐2, silicon nanowires

## Abstract

The recent SARS‐CoV‐2 pandemic has highlighted the urgent need for novel point‐of‐care devices to be promptly used for a rapid and reliable large screening analysis of several biomarkers like genetic sequences and antibodies. Currently, one of the main limitations of rapid tests is the high percentage of false negatives in the presence of variants and, in particular for the Omicron one. We demonstrate in this work the detection of SARS‐CoV‐2 and the Omicron variant with a cost‐effective silicon nanosensor enabling high sensitivity, selectivity, and fast response. We have shown that a silicon (Si) nanowires (NW) platform detects both Sars‐CoV‐2 and its Omicron variant with a limit of detection (LoD) of four effective copies (cps), without any amplification of the genome, and with high selectivity. This ultrasensitive detection of 4 cps allows to obtain an extremely early diagnosis paving the way for efficient and widespread tracking. The sensor is made with industrially compatible techniques, which in perspective may allow easy and cost‐effective industrialization.

## Graphical abstract

Synthetic SARS‐CoV‐2 and Omicron variant detection with a cost‐effective silicon nanowire sensor has been demonstrated with high sensitivity, selectivity, and a fast response. The sensor reaches a 4 cps limit of detection (LoD), even for the Omicron variant, without any genome amplification, and with strong selectivity. The platform is made with industrially compatible techniques, which in perspective may allow a widespread diffusion.

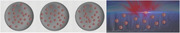

## 1 INTRODUCTION

The SARS‐CoV‐2 pandemic has shown a strong demand for novel cost‐effective and portable devices for fast and accurate analyses. Rapid and widely diffused COVID‐19 diagnostic tests have emerged as a priority for clinical management and outbreak control.^[^ ^1^ ^]^ Looking to the emergency management, as in the case of the Italian surveillance system recognized as one of the most successful models, it emerges a consistent data collection of clinical services and investigated cases across the whole country.^[^ ^1^, ^2^ ^]^ In this case, a deep and spread data collection has been used to anticipate a possible disease outbreak for an efficient contagions control. Another proof of the importance of a strategic data analysis to operate mitigation and diffusion control strategies is represented by the remarkable system adopted in South Korea.^[^ ^3^, ^4^ ^]^ All the successfully employed strategies have in common a constant analysis of the pandemic diffusion making critical the use of rapid, large scale feasible, and reliable COVID‐19 tests. However, the commercially available tests for COVID‐19 fail in the coupling of cost‐effective spread analyses with strong reliability.

During the COVID‐19 emergency, the main detection approach was based on RNA detection by Nucleic Acid Amplification Tests (NAAT) and antigen tests using lateral flow technology.^[^ ^2^, ^5^, ^6^, ^7^, ^8^, ^9^, ^10^ ^]^

The NAAT procedure uses a quantitative polymerase chain reaction (PCR) and it is much more sensitive than antigenic tests. Real‐time reverse‐transcription PCR (rRT‐PCR) is actually considered the gold standard for a reliable genome analysis and recognized by the World Health Organization (WHO) as the routine analysis for SARS‐CoV‐2 case confirmation.^[^ ^2^, ^11^ ^]^ For a long time and in several countries rRT‐PCR assays were the only tests considered reliable for the COVID‐19 diagnosis, while antigenic assays (lateral flow tests) were used as a first screening before a confirmatory test with NAAT. However, NAAT approaches commonly require several hours to get results (>2–4 hours), so that this led to one or more days for the diagnostic outcome for the patient during the pandemic situation. Moreover, PCR requires an expensive set‐up, expert personnel, and a specialized operating laboratory. All these points strongly limited the possibility of a large screening by NAAT as directly experienced in these years and pushed the adoption of other sensing strategies.

The other SARS‐CoV‐2 diagnostic method was the serological analysis. In this case, the biomolecular targets of the assay are the antibodies related to the SARS‐CoV‐2 disease (IgM and IgG antibodies) present in the patient's serum.^[^ ^12^ ^]^ These tests are faster and cheaper than a NAAT test but are considered less reliable. A strong intrinsic issue of these assays is the time required for the antibodies formation that commonly happens in the body after an incubation period of 5 days with their increment from the 5^th^ to the 15^th^ day of disease.^[^ ^13^ ^]^ This makes these tests not suitable for an early COVID‐19 screening when the patient becomes infective around the third day; moreover, he can infect others even before the presence of these antibodies in his blood (with false negative recognition by these tests).^[^ ^13^, ^14^ ^]^ Hence, serological assays are used as a retrospective assessment of the disease.^[^ ^2^ ^]^ Following the WHO guidelines, in cases of NAAT negative results in opposition to strong suggestions of a COVID‐19 infection, a serum analysis performed during different phases can permit to validate the presence of the disease. Hence, a serological analysis is a strong instrument mostly for a retrospective assessment but a time window of false negative limits its reliability.

NAAT and serological analyses were important instruments during the emergency, however, the real game‐changing has been the introduction and spread of rapid antigen tests (also known as lateral flow tests).^[^ ^10^ ^]^ Lateral flow tests are antigen tests based on the detection of viral proteins and can be performed directly on biological samples, commonly from nasopharyngeal swabs. In the first phase of the emergency, the withdrawal of nasopharyngeal samples was restricted to the only specialized personnel in biochemical laboratories. Following the proceeding of the emergency and increasing the number of portable cost‐effective lateral flow tests, different countries adopted these assays as a first screening (as in Italy) or in some cases even as a substitute for NAAT procedures (as in the UK) with cheap measure kits commercially available for the use by the patient itself. The reliability of these tests strongly depends on the swab withdrawal that can have a detrimental effect on the result causing a false negative.

An additional important reliability issue for the SARS‐Cov‐2 diagnosis has been determined by the diffusion of different virus variants that could not be detected by the lateral flow tests, especially for a low virus load (considering PCR, Ct values > 25 (cycle threshold value, indicating how much virus an infected person harbors)).^[^ ^15^, ^16^ ^]^ Indeed, false negative or in general an impaired detection of the omicron variant by rapid antigen tests has been reported. Ostermann et al. reported a rate of true positive results for omicron samples ranging between 31% and 78% for high virus load (Ct values < 25), while this rate dropped down to 0–8% for lower virus load (Ct values > 25).^[^ ^15^ ^]^ Bayart et al. studied the performances of six SARS‐CoV‐2 different commercial tests to the omicron variant confirming poor performances and a drop of positive results for low viral load down to 0–23% range.^[^ ^16^ ^]^

All these studies demonstrated that lateral flow test detection can have a significant lowering of reliability in case of certain variants of concerns (VoC) as the Omicron one. Actually, the emergence of new VoC is associated with the presence of various mutations that may affect the nucleocapsid protein weakening the performance of lateral flow tests. This represents a critical limitation on the possible use of only lateral flow tests to understand and monitor the pandemic diffusion.

Therefore, for an efficient surveillance system, a rapid and reliable large acquisition of clinical results is extremely important. A delay of or the loss of reliability in the analyses causes a lowering in the efficiency of treatment decisions enhancing the disease spread and compromising contact tracing strategies. Larremore et al. have modeled how an increase of the speed of point‐of‐care testing would have a significantly greater impact on fighting back the spread of SARS‐CoV‐2 compared to an improvement of the sensor limit of detection (LoD) performance.^[^ ^17^ ^]^

The evidence given by the experience of SARS‐CoV‐2 emergency made evident how the transmission mitigation is one of the most effective strategies for a real outbreak control. The access to rapid and large diffuse analyses can have a terrific impact on the pandemic diffusion control and quelling.^[^ ^18^, ^19^, ^20^ ^]^ Unfortunately, all the assays available in the markets present several drawbacks, and an urgent demand for novel sensing platforms able to combine the reliability of NAAT with the rapid and spread testing capability of lateral flow test is still present. Most of the scientific community focuses its effort on the research of novel sensors able to guarantee rapidity, low‐cost fabrication, portability, reliability, sensitivity, and selectivity. In this context, the realization of innovative point of care (PoC) devices that respond to all these performances and characteristics has been one of the most discussed challenges in these decades and a major priority in the “Grand Challenges for Global Health.”^[^ ^21^, ^22^, ^23^, ^24^ ^]^ The idea of PoC is to move the screening as close as possible to the patient limiting cost with respect to common standard analysis and reducing the hospital time. Moreover, PoC can have a huge impact in developing economies and third‐world countries where specialized bio‐infrastructures are scarcely diffused and suffer several limitations in terms of cost constraints, power supply, and even poor hygienic conditions. Among all the biochemical strategies suitable for PoC device, PCR free methods has been extensively investigated in the last years.^[^ ^25^, ^26^ ^]^ However, the direct detection (label‐free) of the biomolecule target is the most intriguing since it relies on the direct detection of the untreated analyte solution by a functionalized sensing interface without any amplification step and transduction label.^[^ ^27^, ^28^ ^]^

Nanostructures arose as ideal candidates for sensor applications and innovative PoC due to their outstanding physical properties, a size comparable to the target, and high surface/volume ratio making nanotechnology a strategic tool to overcome standard sensor limits ^[^ ^29^, ^30^, ^31^, ^32^ ^]^ and for novel portable and easy‐to‐use platforms.^[^ ^21^, ^22^, ^33^ ^]^ For the detection of SARS‐CoV‐2, several nanomaterials‐based sensors were proposed in these years and plasmonic‐based colorimetric assays were one of the best diffused.^[^ ^12^, ^34^, ^35^, ^36^ ^]^ As an example, Moitra et al. developed a colorimetric assay based on Au nanoparticles (NPs) for the detection of SARS‐CoV‐2‐nucleocapsid‐phosphoprotein obtaining an LoD of 4 nM, and the virus detection in 10 minutes.^[^ ^34^ ^]^ Such Au NP sensors are promising thanks to their rapid and portable analyses. However, the viral load quantity can impact their performances as happens for lateral flow tests.

Qiu and coauthors have proposed a plasmonic biosensor based on localized surface plasmon resonance (LSPR) that takes advantage of the plasmonic photothermal effect to obtain an opportune in situ hybridization temperature and enhance the sensor performances.

This dual‐functional LSPR biosensor proved a high selectivity for the SARS‐CoV‐2 protein, with an interesting detection limit of 0.22 pM.^[^ ^37^ ^]^

A graphene‐based field‐effect‐transistor (FET) has been designed by Seo et al. as an ultrasensitive biosensor for rapid and reliable detection of the SARS‐CoV‐2 in clinical samples.^[^ ^38^ ^]^ The sensor was functionalized with a specific antibody against SARS‐CoV‐2 spike protein with fg/mL LoD for the antibody in buffer and reaching 2.42 × 10^2^ cps mL^−1^ for clinical samples.

As far as a commercial transfer is concerned and especially for a sensor that is supposed to be developed for a large market and a spread use, the industrial compatibility of materials and processes should be considered a priority. Indeed, incompatible materials and approaches with the technology industry is a common drawback for several sensing platforms proposed in the literature to be technological transferred for commercial exploitation. In this context, silicon‐based platforms present a competitive advantages respect others solution since, as well known, silicon is the leading material of microelectronics. This leads to the realization of Si‐based sensors of great commercial interest to limit the production costs thanks to an already widely developed technology.

Among the transduction strategies for sensing, optical methods are the most diffused in several standard approaches (e.g., ELISA, rRT‐PCR, immunoturbidimetry, nanoparticle tracking analysis, and so on), due to their excellent sensitivity, selectivity, and durability.^[^ ^39^, ^40^ ^]^

Silicon (Si) is an indirect bandgap semiconductor, and photoluminescence (PL) at room temperature (RT) requires quantum‐confinement suitable dimensions. Si nanostructures that emit PL at RT appear as extremely promising materials thanks to their optical properties that may offer most innovative and interesting methods of detection. Nanostructures with quantum‐confinement, such as porous Si (pSi) and Si nanocrystals (NCs), have been already widely explored and reported in the literature as an efficient strategy to obtain light from this indirect bandgap semiconductor. Although appealing, both pSi and NCs are characterized by poor mechanical resistance and high luminescence instability with time.^[^ ^41^, ^42^, ^43^ ^]^ These drawbacks represent severe limitations for pSi and NCs implementation in applicative optical sensing devices. 1D materials such as Si NWs offer a huge surface exposed, easy electric pumping, robustness, and compatibility with the current flat technology on wafers.^[^ ^44^, ^45^, ^46^, ^47^ ^]^ Most of the Si NW‐based sensors are based on electrical transduction in a field‐effect transistor configuration requiring state of art fabrication (e.g., nanoscale throughput lithography) and electrical addressing.^[^ ^48^, ^49^, ^50^, ^51^ ^]^ Indeed, PL at RT obtained by quantum confinement is scarcely reported for Si NWs due to the difficulty of obtaining diameters below 10 nm with traditional techniques.^[^ ^46^, ^52^, ^53^ ^]^ Our group already faced this challenge by demonstrating a low‐cost and industrially compatible synthesis of NWs^[^ ^54^ ^]^ with the observation of PL at RT. The obtained PL is intense and stable compared to other nanostructures such as quantum dots and porous Si.

Recently, we have demonstrated the application of these RT luminescent Si NWs as a novel sensing platform.^[^ ^49^, ^55^ ^]^ In particular, label‐free Si NW optical sensors have proved high selectivity (by proper functionalization protocols) and remarkable LoD for proteins, DNA, and exosomes, overcoming the standard detection methods by several orders of magnitude.^[^ ^28^, ^56^, ^57^ ^]^ All these results have made extremely appealing the realization of a luminescent sensor based on Si NW for SARS‐CoV‐2 able to guarantee a rapid and consistent analysis.

In this work, the industrial compatible fabrication of reliable label‐free optical sensors based on Si NWs for SARS‐COV‐2 detection is reported. In particular, we will demonstrate a viral RNA genome detection with a remarkable LOD on the same order of NAAT methods as PCR but without any amplification of the RNA. Moreover, these Si NW sensors are perfectly able to detect the Omicron variant as well as the standard SARS‐CoV‐2 RNA, paving the way to a novel rapid, sensitive, and selective detection strategy that couples the advantage of NAAT accuracy with a rapidity and an easy‐to‐use format comparable to the lateral flow antigen tests.

## 2 RESULTS AND DISCUSSION

To perform the analysis in a limited well‐defined area a lithographic mask process was carried out patterning the wafer with small circular (300 µm in diameter) uncovered silicon areas as demonstrated before.^[^ ^57^ ^]^ This approach is also adopted thanks to the future perspective of a multi‐probe array fabrication that can be enabled by nanoliter spotting equipment for very precise liquid sample confinement on the chip.

**FIGURE 1:**
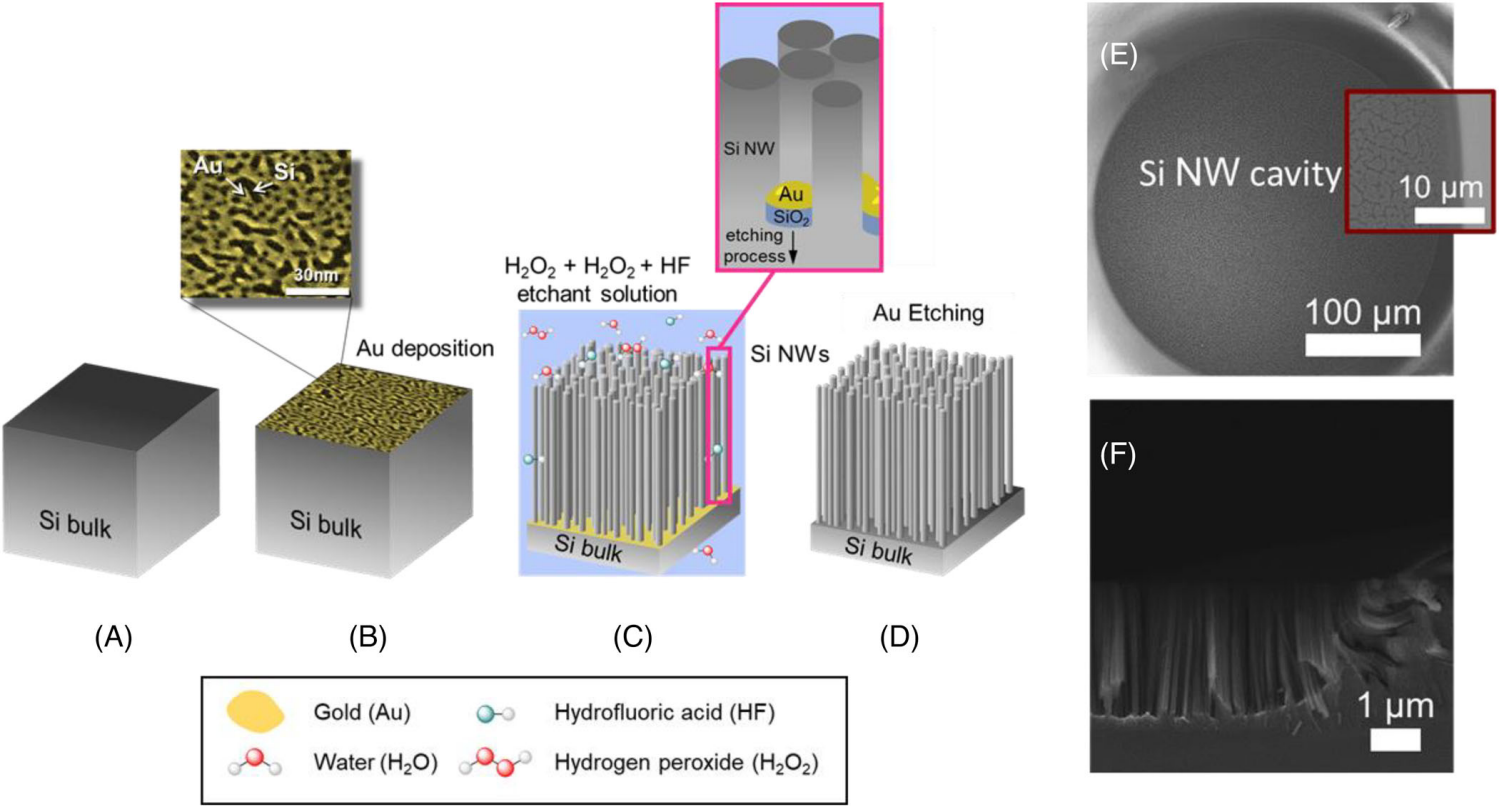
Summary of the thin metal film Metal‐Assisted Chemical Etching for the realization of Si NWs. A, Silicon wafer after the cleaning steps. B, Electron beam evaporation of 2 nm thick discontinuous Au and colored plan view of the deposition as inset. C, Wet etching in H2O2 and HF aqueous solution with details of the etching in the inset. D, Gold etching to remove the Au layer and final sample. E, Top view SEM image of the Si NW cavity designed with a magnification of the border in the inset. F, Cross‐section SEM image of the Si NW cavity

After the lithography process, Si NWs were fabricated in the open Si circular spot by using the thin film Metal‐Assisted Chemical Etching schematized in Figure 1A–D. Patterned Si wafers were cleaned in 2‐propanol and water, etched in aqueous HF solution to remove the native oxide, and then rinsed under nitrogen flux at the end of the process (Figure 1A). A 2 nm thick percolative gold layer is then deposited at RT by an electron beam evaporator (Figure 1B). As seen in the inset to Figure 1B by the colored SEM, the gold layer is discontinuous with nanometric uncovered areas of silicon. Thanks to the photolithographic mask the Au layer was deposited in contact with the silicon only in the circular spot. After the metal deposition, the samples were soaked in a H_2_O_2_ and HF aqueous solution (Figure 1C). The gold acts as a catalyzing agent favoring silicon oxidation underneath it by the presence of H_2_O_2_. The silicon oxide that forms in the metal covered Si region is then etched by the presence of the HF causing an anisotropic etching under the gold and the formation of Si NWs in the uncovered regions as depicted in the inset to Figure 1C. After this step, the gold at the bottom of the fabricated Si NWs is completely removed by a gold etching process. All the etching process is performed at RT and no gold contamination is attested.

The Si NW cavities realized at the end of the fabrication are reported as detected by Scanning Electron Microscopy (SEM) in top view and cross‐section in Figure 1E and F, respectively. A magnification of the border area is reported as the inset to Figure 1E, showing a good Si NW uniformity. In Figure 1F, the cross‐section shows the profile of the cavity border with the typical wall‐effect on the Si NW growth in that region (about 2–3 µm wide) of the photoresist mask, controlled by solution diffusive processes. However, besides the border region (about 2% of the cavity diameter), a narrowly distributed length of about 2.5 µm Si NWs was observed.

**FIGURE 2:**
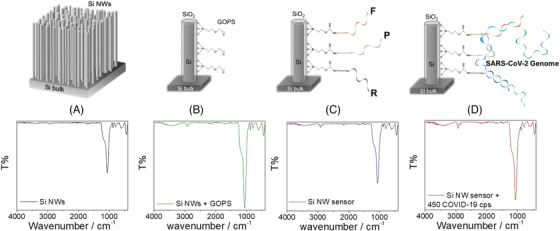
Starting from (A) to (D) the schematic representation of the functionalization protocol is reported. A, As‐prepared Si NWs sample. B, Chemical modification of the surface through GOPS. In (C) the Si NW sensor after the primers (F and R for forward and reverse, respectively) and probe (P) anchoring functionalization is shown. D, Testing of the sample with SARS‐CoV‐2 Genome. At the bottom of each functionalization step, the relative IR spectrum acquired by ATR‐IR is reported

An ad‐hoc protocol for the functionalization of the (surface of the) silicon nanowires was adopted to make the sensor highly selective for the detection of SARS‐CoV‐2. Si NW samples were first cleaned with 2‐propanol and deionized water and rinsed under nitrogen flux (Figure 2A). A silane treatment was then carried out to chemically modify the Si NW surface (Figure 2B). (3‐Glycidyloxypropyl)trimethoxysilane (GOPS) silane was used to produce an epoxy‐terminated Si NWs surface to covalently bound amino‐terminated ss‐DNA oligonucleotides. A silane surface functionalization by GOPS has been preferred since it is a well‐established silicon industry approach which guarantees a stable functionalization.^[^ ^28^, ^58^ ^]^ Once treated with GOPS, Si NWs were functionalized with three ammino‐terminated ss‐DNA oligonucleotides (25 m in length) complementary to the RNA genome sequence of SARS‐CoV‐2 (Figure 2C). This step was performed by dipping the Si NWs sample into 10 µM oligonucleotide solution and then, incubating it at 20°C for 4 hours. The samples obtained after this step represent our sensing platforms.

Lastly, the Si NWs have been treated for the final hybridization step by using the SARS‐CoV‐2 target genome (both synthetic control and Omicron variant) with 4, 40, 400, and 4000 copies (cps) in a 0.01 M PBS at pH 5.5 300 µL solution. The samples immersed in the viral RNA solutions were incubated at 50°C for 4 hours. After each incubation step, the Si NWs were washed with the same buffer used for the incubation and let dry under nitrogen flux.

To demonstrate the functionalization process of the Si NWs measurements of Attenuated Total Reflection in the Infrared range (ATR‐IR) were performed.

IR spectra relevant to pristine Si NWs (black curve) and after different functionalization steps are shown in Figure 2. Si NWs (Figure 2A) present a surface oxide layer, as demonstrated by the band identified at ≈ 1066 cm^−1^ with a small shoulder at ≈ 1200 cm^−1^, and the third signal at ≈ 800 cm^−1^. The first two represent the Si‐O asymmetric stretch (AS) modes and the third is attributed to the symmetric stretch, in agreement with the literature.^[^ ^59^ ^]^ In this case, a silane treatment was performed with GOPS. This step (Figure 2B) led to a significant reduction in the T% for Si‐O asymmetric and symmetric stretching modes suggesting that the alkylsilane molecules are covalently attached, moreover, the appearance of a broad band around 3390 cm^−1^ (OH stretching) and two weak bands relevant to asymmetric and symmetric C‐H stretching at ≈ 2927 and 2857 cm^−1^, attributed to alkyl chain of the silane precursor, confirmed NWs surface modification (green curve).

The blue line plot in Figure 2C represents the IR spectrum of Si NWs after silane treatment and primer anchoring. Proofs of successful anchoring can be found in the decrease in T% of C‐H stretching modes, also shifted to ≈ 2922 and 2852 cm^−1^. Unfortunately, the main peaks demonstrating the presence of nucleic acid anchoring, which fall in the 800–760 cm^−1^ region (out‐of‐plane base vibrations) and between 800 and 880 cm^−1^ (sugar backbone vibrations),^[^ ^60^ ^]^ cannot be discriminated considering the significant overlap with other signals from the substrate. The stretching modes of the PO_2_ group related to the phosphodiester backbone (1080, 1230 cm^−1^) could not be identified unequivocally due to the Si‐O stretching bands. However, it can be observed that the minimum at about 1066 cm^−1^ is particularly less intense than after the silane treatment whereas the shoulder at about 1200 cm^−1^ is more pronounced, probably due to a contribution of the PO_2_ group.

After incubation with the target RNA (Figure 2D), different features appear in the FTIR spectrum. First, the highest decrease in the intensity of the alkyl chain and hydroxyl stretching bands is observed. Moreover, the shoulder at 1200 cm^−1^ is also pronounced and is additionally present a second shoulder at about 950 cm^−1^, ascribable to ribose‐phosphate main chain vibrations^[^ ^61^ ^]^ as a marker for the presence of RNA.

**FIGURE 3:**
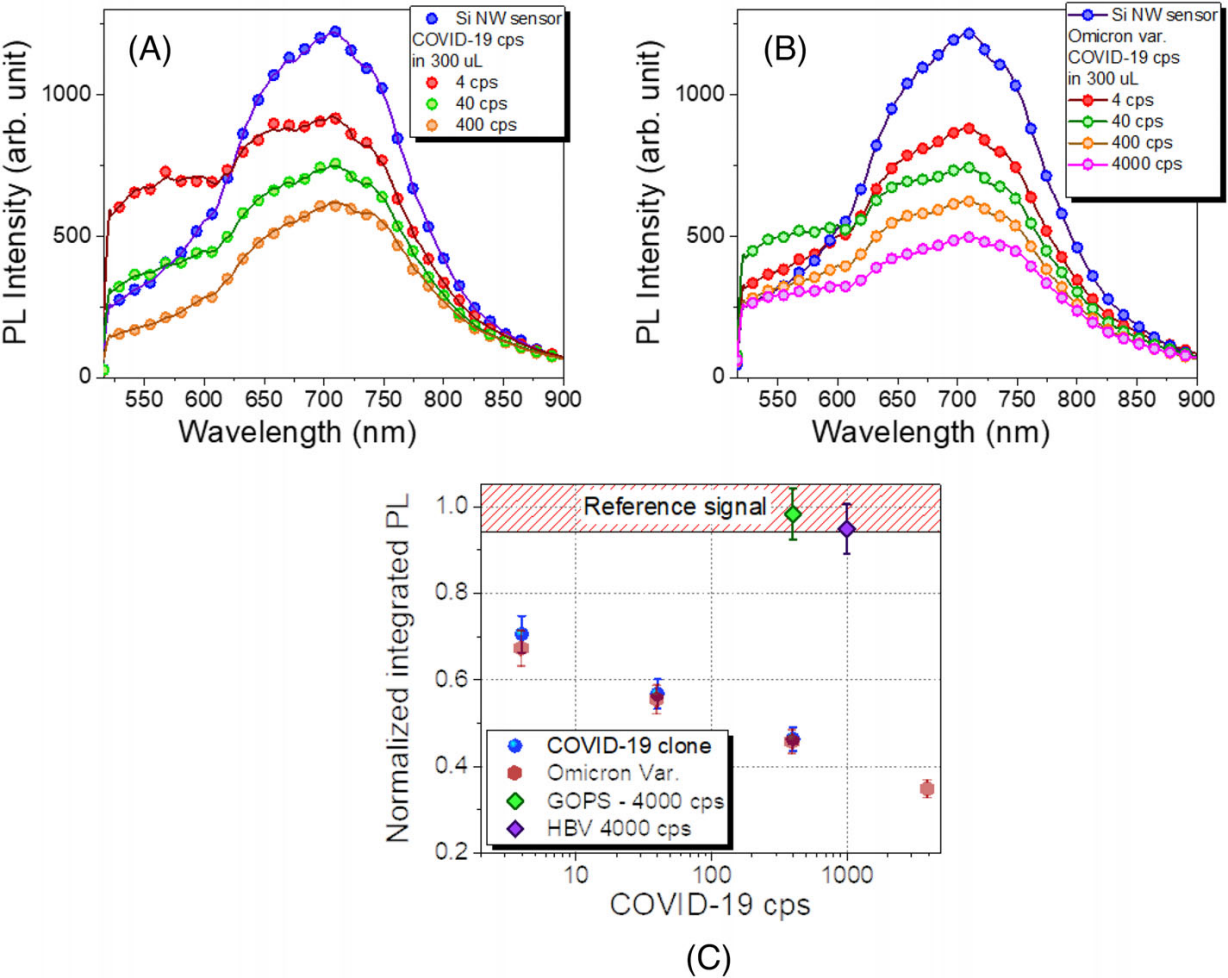
RT PL spectra of the Si NW sensor tested with standard SARS‐CoV‐2and with the omicron variant are reported in (A) and (B), respectively. A, RT PL acquired by the Si NW sensor tested with 4, 40, and 400 cps standard SARS‐CoV‐2. B, RT PL acquired by the Si NW sensor tested with 4, 40, 400, and 4000 cps Omicron variant of the SARS‐CoV‐2. C, Calibration curve obtained as the Normalized PL intensity as a function of the SARS‐CoV‐2 cps. The PL intensity is the Area of the Si NW PL Gaussian fit and it is normalized to the value obtained by the sensor in the same matrix but without any RNA

Once the proper operation of the functionalization protocol was attested by the ATR‐IR measurements, the RT PL of the sensors tested with different amounts of SARS‐CoV‐2 were acquired. In particular, the RT PL analysis of the synthetic SARS‐CoV‐2 genome is shown in Figure 3A, while the Omicron tests are reported in Figure 3B. In both Figure 3A and B, the signal of the sensor without being exposed to any RNA is reported in blue and plays the role of the signal reference. An increase in the viral RNA cps produces a decrease in the PL signal of the sensors as previously demonstrated for this class of platform as the sensing mechanism. The signal variation of the PL compared to the reference for all the SARS‐CoV‐2 genome concentrations both for the synthetic and for the Omicron variants is evident. Moreover, it is worth noting how the sensor without any type of RNA amplification clearly detects down to 4 SARS‐CoV‐2 cps, as can be observed in Figure 3A and B.

These PL measurements were used to build the calibration curve reported in Figure 3C. In this figure, the normalized PL intensity of the sensor is reported as a function of the number of SARS‐CoV‐2 cps. In more detail, the PL intensity for each number of cps is found as the area obtained by the Gaussian fit of the Si NW emission peak and is normalized to the PL intensity (obtained in the same way) of the reference (sensor signal without RNAs). This phenomenon of PL quenching as sensing mechanism was already observed and demonstrated in our previous works on C‐reactive protein and Hepatitis B Virus sensing by Si NWs.^[^ ^28^, ^56^ ^]^ The results obtained by the synthetic SARS‐CoV‐2 are reported as blue dots while the ones obtained by the omicron variant are reported as red hexagons. All the shown errors in this graph were obtained as the standard deviation of different measures (more than five) on different points of the samples and even on different platforms (at least on three different substrates). For both synthetic SARS‐CoV‐2 and Omicron a LoD of 4 cps was demonstrated with a remarkable LoD comparable to NAAT. The sensor response for the value of 4 cps demonstrates how with this approach it is possible to reach the same performances of the PCR without the need of a specialized biochemical facility, and without being impaired by the Omicron variant as happens for lateral flow tests.^[^ ^15^, ^16^ ^]^

However, as described in the introduction what can be a real game‐changing for a pandemic emergency control is rapid and reliable sensing. In the case of synthetic SARS‐CoV‐2 RNA (blue dots, Figure 3C), with respect to the reference signal a 33, 44, and 54% of PL quenching were observed in presence of 4, 40, and 400 cps respectively. For the real Omicron variant analysis, a 33, 45, and 55% PL decrease were observed for 4, 40, and 400 cps, respectively. Considering the standard deviation (about ± 3–4%) the quenching observed for the synthetic RNA was the same observed for the Omicron variant. As expected by the adopted functionalization, a negligible variation in the measure of the same amount of RNA has been obtained for the synthetic genome of the standard SARS‐CoV‐2 compared to the real omicron variant. This is a strong demonstration of reliability and selectivity of the sensing platform for the COVID‐19 independently of the VoC and, as long as the genome sequence complementary to primers and probe remain the same (otherwise the functionalization can be corrected accordingly without any issues). For the Omicron case, also a 4000 cps measurement was carried out obtaining a 65% PL quenching. In the case of COVID‐19 infection with the withdrawal of a biological sample from a swab about 10^5–^10^8^ cps are obtained.^[^ ^62^ ^]^ This number of cps is at least two orders of magnitude higher than the one producing about 65% signal variation in our platform strongly demonstrating the suitability of this sensor for SARS‐CoV‐2 detection. For a commercial disposable platform, the reliability of the measure is the most important parameter, and to further prove the reliability and selectivity of this Si NW sensor, we perform two negative tests. In the first one, Si NW samples were just silane‐treated with GOPS and tested in the SARS‐CoV‐2 detection (4000 cps, green rhombus in Figure 3C) without the use of the primers and probe. The PL quenching obtained was negligible compared to the sensor reference demonstrating that in our procedure the sensing is obtained by the specific primers and probes and not by random physisorption of molecules. Moreover, in the second test, the Si NW sensors after functionalization were then tested with 4000 cps of Hepatitis B Virus (HBV) instead of SARS‐CoV‐2. In this case, an insignificant signal variation compared to the reference was observed. These negative tests strongly proved the high selectivity of the proposed Si NW sensing platform.

## 3 CONCLUSION

In this paper, we demonstrated the detection of SARS‐CoV‐2 and its Omicron variant with very high selectivity and rapid detection, without any amplification reaction of their RNA genome sequences, by a low‐cost label‐free optical sensor based on Si NWs. Moreover, the detection of few cps (down to 4 cps) of SARS‐CoV‐2 and its Omicron variant proved that it is possible to obtain a reliable detection among variants of interest that make rapid flow tests not reliable for low virus load. This result also opens the perspective of an extremely early tracking of the infection and may represent a breakthrough in rapid testing strategies for different variants of the virus.

## 4 EXPERIMENTAL SECTION

### 4.1 Materials

Commercial 4″ silicon wafer (100)‐oriented, n‐type (resistivity ∼ 1–5 Ω·cm) and 500 µm of thickness were purchased from University Wafer. Hydrofluoric acid (HF) 40%, gold etchant, 2‐Propanol (ACS reagent grade), Acetone (ACS reagent grade), PBS tablets were bought from Honeywell while Merk (Sigma–Aldrich) supplied the hydrogen peroxide (H_2_O_2_). The HF/ H_2_O_2_ aqueous solutions used for MACE were prepared by using Milli‐Q Deionized water (resistivity ∼ 18 MΩ·cm). Inactivated SARS‐CoV‐2 virus from Amplirun® Total SARS‐CoV‐2 Control (Vircell Molecular, Granada, Spain) was used for the calibration curve. Primer and probe sequences (Cov‐Fw‐C6amino: 5′ GAC GTC TAA ACC TAC TAA AGA GG 3′; Cov‐Rev‐C6amino: 5′CCT TGT GTG GTC TGC ATG AGT TTA G 3′; Cov‐prob‐C6amino: 5′ TAA CGT TGT TAG GTA CTC GTC ACG ACT GAG 3′) were synthesized by Metabion International AG (Germany). Omicron RNA genome, provided by the human pathologies and COVID center of the University Hospital of Messina (Policlinico di Messina), was extracted and purified from human swab samples using the Maxwell® RSC automated RNA extractor (Promega, USA), accordingly to the procedure reported in the supporting information.

### 4.2 Si NW fabrication

For the Si NW cavity fabrication, a lithographic approach was carried out before the MACE synthesis. Silicon wafers were cleaned following a standard microelectronics‐grade cleaning with a sonic bath in acetone, 2‐propanol (IPA), and Milli‐Q water, followed by a hotplate de‐hydration at 120°C for 600 seconds. After this procedure, a negative resist (AZ5214) was deposited obtaining a layer with a final thickness of about 1.4 µm. A 365 nm optical lithography process by a Karl Suss MA 6 in hard contact mode was then carried out. After the development of the mask, a hard bake step for 10 minutes at 150°C was performed on a hotplate to increase the mask etching selectivity for the HF etching. The used mask permits the realization of circular patterns with diameters of about 150 µm. Finally, Si NWs were synthesized by MACE using as a substrate the patterned wafers. As already reported in several publications of the authors,^[^ ^54^ ^]^ native SiO_2_ was removed by a 2.5 M HF aqueous etching solution and then a 2 nm of Au discontinuous film was deposited in high‐vacuum conditions (<10^−6^ mbar) and at RT by an Electron Beam Evaporator (EBE, Kenosistec). After the evaporation process, the samples were immersed in a 5 M HF and 0.44 M H_2_O_2_ watery solution. The Au acts as a catalyst improving the oxidation (favored by the presence of H_2_O_2_) of the silicon under the gold. Finally, the formed SiO_2_ is selectively etched by the hydrofluoric acid realizing at the end of the day an anisotropic vertical etching under the gold regions. Hence, Si NWs are fabricated in the uncovered and unetched silicon regions while the gold film is removed by a gold etchant (KI solution). All the etching is performed at RT and previous studies demonstrate the absence of Au contamination inside the silicon.

### 4.3 Sensor functionalization protocol

After the synthesis, the Si NW samples were functionalized by a three‐step protocol.

1. Si NW samples were cleaned for 1 minute in IPA and in Milli‐Q water to remove the alcohol residue and finally dried in a nitrogen flux.

2. The cleaned Si NW samples were then chemically modified by a (3‐Glycidyloxypropyl)trimethoxysilane (GOPS) silane layer. The samples were exposed to 10 mL GOPS at 120°C for 4 hours in a low vacuum chamber (about 200 mbar).

3. After the GOPS process, the samples were functionalized by the following anchoring process. The samples were incubated at 20°C for 4 hours with 40 rpm agitation into a 10 µM solution of the forward and reverse primers and the probe for SARS‐CoV‐2 (suspended in 0.01 M PBS buffer pH 7.4). Finally, the samples were cleaned by soaking in a 0.01 M PBS buffer pH 7.4 (same buffer of the primers and probe solutions) and let dry under a nitrogen atmosphere.

After the oligonucleotides anchoring, the sensor functionalization was reached, and the obtained sensing platforms were tested with the SARS‐CoV‐2 target genome. Si NW sensors were incubated at 50°C for 4 hours with 40 rpm agitation with different SARS‐CoV‐2 (both synthetic control and Omicron variant) at a concentration of 4, 40, 400, and 4000 cps in 0.01 M PBS at pH 5.5. Then, Si NWs were washed with the same buffer used for target dilutions and let dry as described above.

### 4.4 Morphological, structural, and optical characterization

A field‐emission Scanning Electron Microscope (SEM, Zeiss Supra 25) has been used for the morphological and structural characterization of the fabricated Si NWs.

Attenuated total reflectance Infrared (ATR‐IR) spectroscopic analysis was carried out on pristine Si NWs and after each functionalization step. IR spectra were acquired in Transmittance mode on a SpectrumTwo PerkinElmer FT‐IR spectrometer equipped with a diamond crystal (wavelength range 4000–500 cm^−1^, resolution 2 cm^−1^, 32 scans).

The PL spectra were obtained by using a Jobin Yvon Horiba HR 800 equipped with a 600 L mm^−1^ grating, and a Peltier −70°C cooled CCD (Synapse). The measures are carried out in a backscattering configuration with a 100× (NA = 0.9) objective used for both excitation and acquisition. The excitation is obtained by a 476 nm line of an Ar^+^ laser with a power of 100 µW onto the sample plane.

## 5 INSTITUTIONAL REVIEW BOARD STATEMENT

The Institutional Review Board of the University of Messina approved this study under the protocol number 36/20 and in accordance with the tenets of the Declaration of Helsinki.

## 6 INFORMED CONSENT STATEMENT

Informed consent was obtained from each patient. The research was carried out in accordance with the tenets of the Declaration of Helsinki and was approved by the Institutional Review Board of the University of Messina (protocol number 36/20). (see Supporting Information).

## CONFLICT OF INTEREST

The authors declare no conflict of interest.

## Supporting information



Supporting Information

## Data Availability

The data that support the findings of this study are available from the corresponding author upon reasonable request.
