# Supplementary material for: SARS‐CoV‐2 and omicron variant detection with a high selectivity, sensitivity, and low‐cost silicon bio‐nanosensor
Source: Nano Sel. 2022 Dec 29:10.1002/nano.202200188. Online ahead of print. doi: 10.1002/nano.202200188 (PMC9880655; doi:10.1002/nano.202200188)
Supplement: Supplementary file 1 — Supporting Information [file NANO-9999-0-s001.docx]

Supporting Information

SARS-COV-2 OMICRON DETECTION WITH A HIGH SELECTIVITY, SENSITIVITY, AND LOW-COST SILICON BIO-NANOSENSOR

*Antonio Alessio Leonardi^1,2,‡^, Emanuele Luigi Sciuto^3,4,‡^, Maria José Lo Faro^1,2^, Barbara Fazio^3,5,^ Maria Giovanna Rizzo^4^, Giovanna Calabrese^4^, Luca Francioso^5^, Rosanna Picca^6^, Francesco Nastasi^4^, Giuseppe Mancus^7^, Corrado Spinella^3,8^, Wolfgang Knoll^9^, Alessia Irrera^3*^, and Sabrina Conoci^3,4,8,10*^*

1 Dipartimento di Fisica e Astronomia “Ettore Majorana”, Università degli studi di Catania, Via S. Sofia 64, Catania, 95123 Italy

2 CNR-IMM Catania Università, Istituto per la Microelettronica e Microsistemi, Via S. Sofia 64, 95123, Catania, Italy

3 Lab SENS, Beyond NANO, Viale Ferdinando Stagno d’Alcontres 31, Messina, 98166, Italy

4 Dipartimento di Scienze Chimiche, Biologiche, Farmaceutiche, ed Ambientali, Università degli studi di Messina, Viale Ferdinando Stagno d’Alcontres 31, Messina, 98166 Italy

5 CNR-IMM, Istituto per la Microelettronica e Microsistemi, Via Monteroni, University Campus, 73100 Lecce (Italy)

6 Dipartimento di Chimica, Università degli studi di Bari, Via E. Orabona 4, Bari, 70126 Italy

7 Dipartimento di Patologia Umana dell'adulto e dell'età evolutiva Gaetano Barresi, Università degli studi Messina, via Consolare Valeria 1, Gazzi (Me) Italy

8 CNR-IMM Istituto per la Microelettronica e Microsistemi, Zona Industriale, VIII strada 5, Catania, 95121 Italy

9 Department of Scientific Coordination and Management, Danube Private University, A-3500 Krems, Austria

10 Dipartimento di Chimica "G. Ciamician", Università degli studi di Bologna, Bologna, Italy

^‡^ These authors contributed equally to the work

*corresponding authors: AI [irreaalessia@gmail.com](mailto:irreaalessia@gmail.com), SC [sabrina.conoci@unime.it](mailto:sabrina.conoci@unime.it)

*1.1. Standard SARS-CoV-2 RNA sample preparation*

Inactivated SARS-CoV-2 virus from Amplirun® Total SARS-CoV-2 Control (Vircell Molecular, Granada, Spain) was used for safety reasons.

The sample was prepared for the RNA extraction by, first, adding 500 μl of nuclease-free water, for Molecular Biology, to the vial and mixed until completely reconstituted. The concentration was approximately 30,000 copies/ml once reconstituted. Finally, the sample has been vortexed for 30 seconds to dissolve and homogenize completely.

250 μl of the suspension was used to extract viral RNA using the TRIzol reagent (Invitrogen, Carlsbad, CA, USA). All glassware used for the preparation of the samples were RNase, DNase, and protease-free.

Briefly, to isolate the SARS-CoV-2 RNA has been added 0.5 mL of isopropanol to the aqueous phase for 1 mL of TRIzol™ Reagent used for lysis. The sample was centrifuged for 10 minutes at 12,000 × g at 4°C, discarded the supernatant and the pellet was resuspended in 1 mL of 75% ethanol. Then centrifuged for 5 minutes at 7500 × g at 4°C. Finally, the RNA pellet has been resuspended in 30μL of RNase-free water and incubated in heat block at 60°C for 15 minutes.

Before processing the sample for the experiments, the sample concentration and quality was assigned by 260/280 nm and 260/230 nm ratios using the Thermo Scientific NanoDrop 1000 Spectrophotometer, revealing an RNA concentration of approximately 15 copies/µL. Then, serial dilutions of the extracted standard SARS-CoV-2 RNA sample in 0.01M PBS at pH 5.5 have been prepared in order to obtain working solutions containing a total of 4, 40, 400, and 4000 copies (cps) of RNA in 300 µL.

The expression levels of SARS-CoV-2 were amplified by using the following primer sequences:

Cov-Fw-C6amino: 5’ GAC GTC TAA ACC TAC TAA AGA GG 3'; Cov-Rev-C6amino: 5’CCT TGT GTG GTC TGC ATG AGT TTA G 3’; Cov-prob-C6amino: 5’ TAA CGT TGT TAG GTA CTC GTC ACG ACT GAG 3’.

Primers and probe customization was performed as follows. The GISAID databank was used to download the whole SARS-CoV-2 genome sequence [1]. Once imported to the SnapGene software (from Dotmatics, UK), the sequence was used to design primers and probe that were complementary to the ORF N-9b_Nucleocapsid protein coding region, already selected as region of annealing in the most diffused commercial PCR kit for COVID-19 molecular screening. The selectivity of the designed primers towards the Omicron variant genome by using the Clustal Omega tool of EMBL for the sequence alignment [2].

- 1. *Quantification of SARS-CoV-2 Omicron variant RNA copies.*

The quantification of the RNA copies of Sars-CoV-2 Omicron variant, used as target in the Silicon nanowires (SiNWs) sensing tests, has been performed according to the correlation reported in the work of Brandolini et al. (2021) [3].

They proposed a direct comparison between the Ct values obtained by diagnostic routine tests based on qRT-PCR and the total RNA copies/µL estimated by digital PCR (dPCR). 48 Sars-CoV-2 samples at various concentrations have been preliminarily processed, using the Nextractor technology, for the RNA extraction. Subsequently, two aliquots of each RNA sample have been quantified by using dPCR and qRT-PCR amplification performed by the QuantStudio 3D Digital PCR and the Seegene Allplex SARS-CoV-2 technology, respectively. The RNA quantification, then, provided a statistical correlation of the two methods, graphing the absolute concentrations estimated by dPCR, expressed as copies/µL (reported on a log10 scale), as a function of the Ct values (Figure 1).

**Figure 1.** Correlation between the results obtained from the same set of samples by digital polymerase chain reaction (dPCR, number of RNA copies per _L on a log 10 scale) and quantitative reverse transcription polymerase chain reaction (qRT-PCR) with preliminary RNA extraction (Nextractor Ct values).

The equation extrapolated from the graph (Eq.1) has been used, then, to calculate the number of RNA copies of the viral stock sample and its dilutions used for the Si NWs functionalization.

**Eq.1.** y = −0.3062x + 10.50

Considering the Ct=18.6 of stock sample it was estimated a concentration of about 10^4^ copies/µL. Thus, the stock went through 10-fold serial dilutions in 0.01M PBS pH 5.5, in order to obtain working solutions containing 4, 40, 400, and 4000 copies of RNA in a final volume of 400 µL. These solutions were, then, used for the hybridization step in the substrate functionalization.

**References**

1. GISAID - Search Available online: https://www.gisaid.org/help/search/ (accessed on 21 April 2022).

2. Clustal Omega < Multiple Sequence Alignment < EMBL-EBI Available online: https://www.ebi.ac.uk/Tools/msa/clustalo/ (accessed on 21 April 2022).

3. Brandolini, M.; Taddei, F.; Marino, M.M.; Grumiro, L.; Scalcione, A.; Turba, M.E.; Gentilini, F.; Fantini, M.; Zannoli, S.; Dirani, G.; et al. Correlating QRT-PCR, DPCR and Viral Titration for the Identification and Quantification of SARS-CoV-2: A New Approach for Infection Management. *Viruses* **2021**, *13*, 1022, doi:10.3390/v13061022.
